# Building Clinical Clerkships Capacity in a Resource-limited Setting: The Case of the Kilimanjaro Christian Medical University College in Tanzania

**DOI:** 10.29024/aogh.15

**Published:** 2018-04-30

**Authors:** Chrispina N. Tarimo, Gibson E. Kapanda, Charles Muiruri, Ahaz T. Kulanga, Esther Lisasi, Kien A. Mteta, Egbert Kessi, Deodatus Mogella, Maro Venance, Temu Rogers, Lucy Mimano, John A. Bartlett

**Affiliations:** 1 Kilimanjaro Christian Medical University College, Moshi, TZ; 2 Kilimanjaro Christian Medical, Centre Medical Education Partnership Initiative, Durham, North Carolina, US; 3 Kilimanjaro Christian Medical Centre Medical, TZ; 4 Kilimanjaro Christian Medical, Centre Medical Education Partnership Initiative, Moshi, TZ; 5 University of Cape Town, ZA; 6 Medicine, Global Health, and Nursing, Duke University, US

## Abstract

**Background:**

The shortage of medical doctors in sub-Saharan Africa (SSA) has resulted in increased enrolment in medical schools, which has not been matched with increased faculty size or physical infrastructure. This process has led to overcrowding and possibly reduced quality of training. To reduce overcrowding at its teaching hospital, the Kilimanjaro Christian Medical University College introduced eight-week peripheral clerkship rotations in 2012. We explore students’ perceptions and attitudes towards peripheral hospital placements.

**Method:**

The clerkship rotations were conducted in eight hospitals operating in the northern Tanzania, after evaluating each hospitals’ capabilities and establishing the optimum number of students per hospital. Paper-based surveys were conducted after student rotations from 2014 to 2016.

**Results:**

Overall student satisfaction was moderate (strength of consensus measure (sCns), 77%). The three cohorts exhibited improving trends over three years with respect to satisfaction with clinical skills and attitude towards placements. student-preceptor interaction was rated highly (sCns 81–84%). The first cohort students expressed concerns about limited laboratory support, and poor access to Internet and learning resources. Specific interventions were undertaken to address these concerns.

**Conclusions:**

Student experiences in peripheral rotations were positive with adequate satisfaction levels. Opportunities exist for medical schools in SSA to enhance clinical training and relieve overcrowding through peripheral clerkship rotations.

## Background

The scarcity of human resources for health is a global problem, which is further amplified by maldistribution between urban and rural areas within countries [1,2]. In Tanzania, for example, the doctor to population ratio stood at 0.26 per 10,000 in 2012 [3], which is far lower than the recommended 1 to 5,000 [4]. On the other hand, despite the overall low doctor to population ratio, in rural areas, where 75% of the total population lives, only 31% of the available doctors practice in these settings [2]. This shortage impacts health care service provision, especially in places where it is much needed and has greater impact [4].

To reduce healthcare worker shortages in sub-Saharan Africa (SSA), many governments have encouraged the establishment of new medical schools (both public and private) and increasing student enrolment in existing schools. Within a 12-year period (1997 to 2009), Tanzania expanded the enrolment of medical students by five-fold in eight public and private health training institutions [5]. However, this expansion in enrolment has not been accompanied by adequate increases in faculty size or physical infrastructure. The Study on sub-Saharan African Medical Schools identified lack of basic infrastructure (reliable sources of power, teaching laboratories, libraries, teaching space, to mention a few) coupled with critical faculty shortages as impeding factors for the expansion of medical schools in Africa [5]. Consequently, classes were overcrowded, leading to compromise in the quality of training, especially in the clinical clerkships. Furthermore, most of the medical schools in sub-Saharan Africa are located at tertiary hospitals (which also serve as teaching hospitals). These teaching hospitals have limited clinical practice areas and mostly concentrate on curative care [6]. There is, thus, a dire need for innovative approaches to overcome these challenges.

According to a review by Barrett and colleagues, teaching sites outside the tertiary teaching hospitals have been found to be under-utilized. They represent potentially effective areas for medical education in low- and middle-income countries (LMIC) [7], which could solve some of the problems pertinent to increased numbers of medical students. It has been shown that peripheral placements can provide a high-quality clinical learning environment, hands-on practical experience, and at the same time students get acquainted with diseases that are common in communities and that are not referred to the tertiary teaching hospitals [8]. Students get the opportunity to see a wider spectrum of diseases, may have more patient-preceptor interaction, and learn the culture of the communities around the peripheral hospital [9]. Furthermore, peripheral placement inculcates the culture of teamwork within students [10].

Like many other medical schools in SSA which increased enrolment rate over the past five years up to 2010 [1], medical schools in Tanzania faced similar challenges. Thus, in Tanzania with only one medical school enrolling 55 first-year medical students in 1991, by 2015 the 11 Tanzania Commission for Universities (TCU)-accredited medical schools (both public and private) were enrolling a total 1,580 students per year (an average increase of 144 students per school) [11].

In 2010, the United States Government directed unprecedented resources to support African medical schools in meeting their training needs and triggered a number of collaborations, including those between south-south partners in Africa [12,13]. The Medical Education Partnership Initiative (MEPI) distributed $130 million to 13 medical schools in 12 sub-Saharan African countries. The thematic focus of MEPI was on improving the quality, quantity, and in-country retention of medical graduates along with building research capacity in African medical schools [14]. The Kilimanjaro Christian Medical University College (KCMUCo), located in northern Tanzania and one of the 13 MEPI beneficiary African medical schools, was established in 1997 with 15 students. By 2010, when the MEPI project started the number of students, had increased by almost nine-fold to 129 [15]. By 2012 the KCMUCo had an average annual undergraduate medical student population (year 1 through year 5) of 700.

In the first two years of medical education KCMUCo students learn basic sciences, while during the third through fifth year clinical clerkships are core. To cope with the increased number of students, without compromising the scarce training resources (including faculty) during basic sciences years (year 1 and year 2), MEPI program has utilized, with great success, a learning management system (LMS), team-based learning [16] and other technologies to support medical training.

The Kilimanjaro Christian Medical Centre (KCMC), one of four referral hospitals in Tanzania, which has an official bed capacity of 418 in major clinical wards (paediatrics, internal medicine, surgery, obstetrics and gynaecology) (KCMC Annual Report, 2014), is the principal clinical training site for KCMUCo clerkships. In this regard, with an average of 450 medical students in the third to fifth year, the teaching hospital capacity was inadequate to accommodate the increased number of students. To tackle the problem, through MEPI, KCMUCo introduced the eight-week peripheral hospital placement program for third year medical students (MD3) within Kilimanjaro and Arusha regions during the 2012/13 academic year to undertake their junior clerkships. This was aimed at reducing student overcrowding at their main teaching hospital and to expose the students to a real working environment with hands-on practice experience. This was also geared toward training medical students in the rural settings as one of the ways to enhance retention of medical doctors where they are needed most. This study provides a three-year (2014–2016) description of the implementation strategies employed to enhance the quality of clinical clerkships in the resource-limited context through the utilization of peripheral hospitals and to assess students’ perceptions of peripheral hospital clinical clerkships using three different student cohorts.

### Approach

In order for the College to implement the project effectively, during the 2012/2013 academic year a task force comprised of senior administrative/academic officers was formed. It was comprised of the Executive Director of Kilimanjaro Christian Medical Center (KCMC), Provost, Deputy Provost-Academic Affairs, Deputy Provost-Administration, Dean of the Faculty of Medicine, Chairs of Departments or specialists from Internal Medicine, Surgery, Pediatrics, Obstetrics and Gynecology, anaesthesia, and senior nursing officers. The main mandate of the task force was to identify and assess capabilities of peripheral hospitals to train students and recommend appropriate training sites for clerkship rotations. Since KCMC is a referral hospital and offers continuing professional development for most of the peripheral hospital staff, this already existing relationship was used to assist in the selection of training sites. The task force utilized a checklist to assess the peripheral hospitals capabilities, and it included the hospital staffing level (number of specialists, general practitioners and middle cadre staff), operative capacity (number of operating rooms, anaesthesia staff, theatre equipment and operative care services available) and also accommodation for students and supervisors.

The taskforce provisionally identified 19 hospitals operating in the northern zone of Tanzania, which are located within a radius of not more than 170 kilometres with easy road access from KCMUCo. Consultative meetings with District Medical Officers (DMOs) with jurisdiction of the identified hospitals were held to seek clearance in using the hospitals for training purposes. The task force finally settled on eight hospitals, which met the pre-specified standards, and also signed the Memorandum of Understanding (MoU). These included Mawenzi Regional Referral Hospital, St. Joseph Designated Council Hospital, Kibosho Designated Council Hospital, Tanganyika Planting Company (TPC) Hospital, Same Council Hospital and Gonja Hospital in Kilimanjaro region, and St. Elizabeth Hospital and Mt. Meru Regional Referral Hospital in Arusha region. Of these, four hospitals, namely, TPC, Gonja, Kibosho and Same were classified as rural (located in district or small towns – population size of less than 30,000 inhabitants) while the remaining four, as urban (located in big towns or city – population size of 30,000 or more inhabitants). The capacities of the rural and urban peripheral hospitals have been shown elsewhere [17].

After the selection of the hospitals, a one-week workshop focusing on the learning and teaching objectives of the students who will be placed at the hospitals was held at the teaching hospital (KCMC). The participants of the workshop included the medical doctor in-charge/hospital managers, selected preceptors (basing on their roles, experience and qualifications) from the four departments (surgery, obstetrics & gynaecology, paediatrics, and internal medicine) at each of the peripheral hospitals respectively and heads of the departments at the teaching hospital. The organization and logistics of the clinical clerkship rotation were also charted at this workshop.

The eight-week clinical clerkship rotations were carried out at the eight selected peripheral hospitals. A stratified random sampling was used to distribute MD3 students proportionally to the hospital bed capacity (size of the hospital). However, prior to the placement, students participated in 11-week clinical practical skills training (CPST) whereby they rotated in the teaching clinical laboratory, wet laboratory, clinical skills laboratory, multipurpose laboratory, internal medicine report room and dissection hall to orient the students on patient disease diagnosis and care and also application of basic sciences in patient care.

No formal assessment of the rotation was conducted for the 2012/2013 cohort because the intervention had just been rolled out and hence was used as a “pilot”. During their rotation, students carry out junior clerkship on surgery, obstetrics & gynaecology, paediatrics, and internal medicine, each lasting two weeks respectively. During clinical clerkship they collected patient histories, conducted physical examinations, ordered investigations, compiled differential diagnoses, participated in making therapeutic plans, suggested any diseases/situations of preventable caution to each patient and used Rapid Diagnostic Tests (RDTs) and Point of Care (POC) devices. Students were required to fill their logbooks, which were reviewed by their mentors/preceptors during clinical rotations. Furthermore, faculty members from respective departments (that is, surgery, obstetrics & gynaecology, paediatrics, and internal medicine) from KCMC made regular follow-up supervisory visits on daily basis when students are in that rotation. Also, residents from respective departments accompany medical students during rotations to provide on the spot assistance to students when need arises and at the same time the residents get peripheral hospital experience that may motivate them to accept rural deployment.

To facilitate the distribution and follow up of students at the respective peripheral hospitals, the MEPI project sponsored two cars (hard-top Landcruiser and a mini-bus). Also, to overcome some of the challenges arising from preceding rotations, from time-to-time Medical Innovation Program (MIP) write-ups were designed to secure funds from the MEPI project. To ensure sustainability of the program, the KCMUCo has taken over the financing of running costs of the vehicles and scaling-up the activities initiated through MEPI project (provision of RDT and POC devices, and MiFi with internet bundles).

To assess the effectiveness of the peripheral hospital placement in reduction of rural-urban mal-distribution of physicians, KCMUCo has established a graduate tracking system that follows up students after graduation and completion of their internship. This tracking will enable the college to know the proportion of graduates accepting rural practices and their retention in those settings.

### Post-rotation assessments

One week after their return from the peripheral hospital rotations in years 2014 through 2016, repeated cross-sectional surveys using a quantitative approach were completed by the MD3 students. All 147, 153 and 156 MD3 students were eligible to participate in the surveys during the three consecutive years respectively. The data were collected over a period of one day through paper-based surveys whereby students completed self-administered questionnaires. The questions addressing student satisfaction with peripheral hospital placement were analysed in four main domains: (i) general satisfaction with peripheral hospitals placement; (ii) attitudes toward peripheral hospital placement; (iii) satisfaction with clinical practice competencies/skills acquired; and (iv) satisfaction with preceptorship/supervision at the peripheral hospital. The challenges encountered and suggestions for improvement were also accessed. After each round of assessment, prominent challenges that could affect the quality of learning at the peripheral hospital were taken into account for improvement during the forthcoming placement and outcomes evaluated thereafter. For example, during 2014/2015 Rapid Diagnostic Test (RDT) toolkits were piloted through MIP at one urban-located hospital (St. Joseph) and one rural-located hospital (Gonja), in response to the lack of available laboratory testing. Also, Mobile WI-FI (MiFi’s), that is, mobile internet for frequent interface were also distributed by MEPI project to all students in a ratio of 1:10 in response to lack of internet connectivity at the peripheral hospitals. After the rotations, the effect on satisfaction and attitudes towards peripheral hospital placements were assessed by comparing students provided and not provided with RDT toolkits. Also, the frequency of use and usefulness of MiFi’s with suggestions for improvement were assessed after the placement.

Most of the questions were 5-point Likert scale type (strongly agree “5” to strongly disagree “1”). Since no standardized and validated questionnaires were available, the monitoring and evaluation team developed a questionnaire that was piloted with 2013 MD3 students and showed high reliability with Cronbach’s Alpha of 0.949.

The data collected were entered into a computer using Statistical Package for Social Sciences (SPSS) version 20.0 computer program. After data cleaning, data were summarized using descriptive statistics, frequency distributions and strength of consensus measure (sCns). The strength of consensus measure was preferred over use of descriptive statistics because it objectively measures the strength of group agreement or disagreement to constructs. A strength of consensus measure of at least 80% was considered strong. Tests of significance were done using Mann-Whitney U test at 5% level of significance. A p-value < 0.05 was considered significant.

## Results

**Figure 1:**
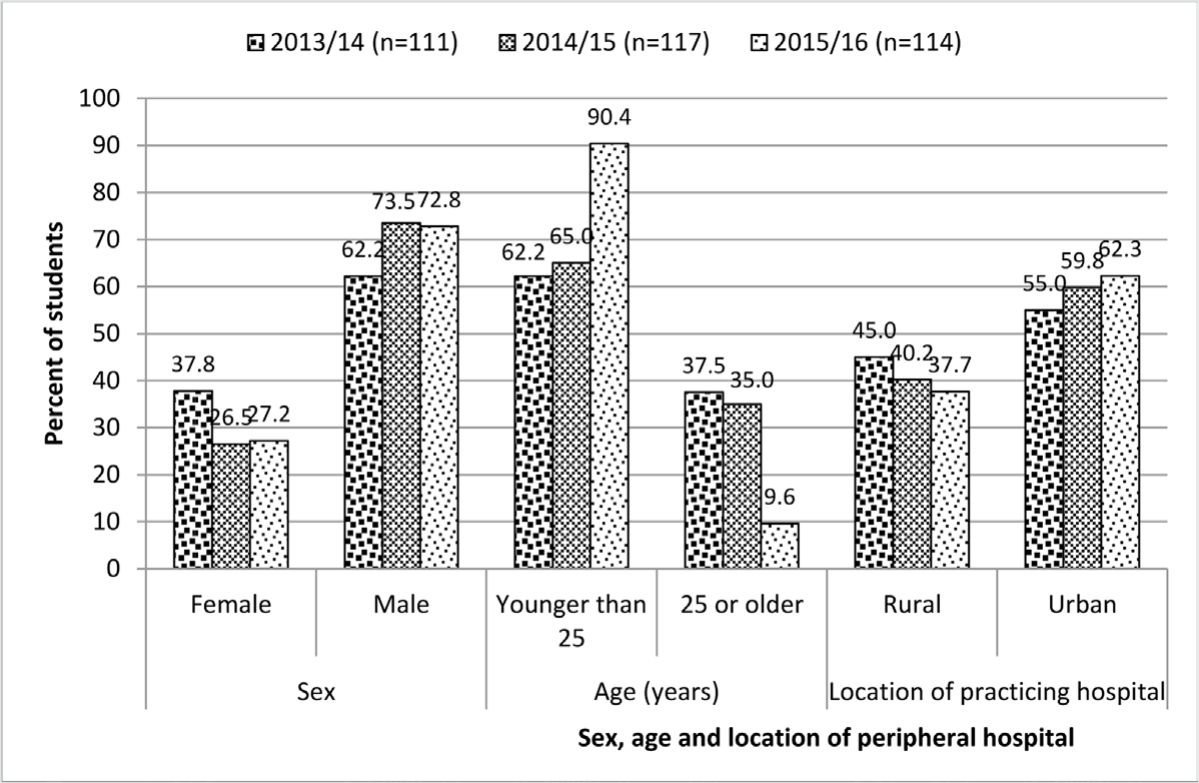
Distribution of students according to sex, age and location of placement peripheral hospital.

Seventy-five percent (342 out of 456) of MD3 students took part in the three consecutive surveys. The majority were male (69.6%), younger than 25 years (72.5%) and placed in peripheral hospitals located in urban centres (59.1%). The yearly distribution of students according sex, age and location of peripheral hospital is shown in Figure 1.

**Figure 2:**
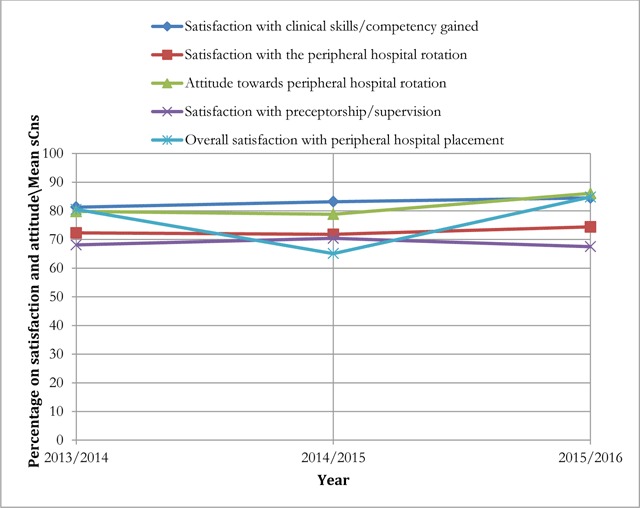
Three-year trend on satisfaction and attitude towards peripheral hospital placement. sCns = Strength of consensus measure.

Out of 342 MD3 students who completed surveys, 319 (93.3%) answered the question on overall satisfaction with peripheral hospital placement. Of these 245 (76.8%) were satisfied with the rotation with strength of consensus measure (sCns) of 76.8%. The overall satisfaction exhibited a declining trend in strength of consensus measure (from 80.5% in 2013/2014 to 65.1% in 2014/2015) and then in 2015/2016 rose to 84.8%. Analysis of different domains probed for satisfaction indicated that, satisfaction with clinical practice competencies/skills acquired increased in consecutive cohorts (sCns rising from 81.3% in 2013/2014 to 84.5% in 2015/2016) indicating strong agreement. Though slightly declining in 2014/2015, attitude towards peripheral hospital placement in general showed a rising trend, which was also indicative of strongly positive attitudes. Similar to attitude, general satisfaction with peripheral hospital rotation also exhibited a rising general trend though with weak strength of consensus measure (sCns < 80%). On the other hand, satisfaction with preceptors/supervisors at the peripheral hospital, while slightly rising in 2014/2015 to sCns = 70.4% from sCns = 68.1% in 2013/2014, it declined to 67.5% in 2015/2016 showing weak agreement on satisfaction with preceptors/supervisors (Figure 2).

A detailed analysis was performed on the three-year trends of strength of consensus to specific constructs related to general satisfaction with peripheral hospital placement and attitude towards peripheral hospital placement. Regarding general satisfaction with peripheral hospital placement, some constructs exhibited increasing trends through the years; others a fluctuating trend, and one construct showed a declining trend. Increasing trends were noted for constructs related to clear initial orientation prior to placement; learning opportunities being consistent with learning goals; availability of opportunities to apply basic sciences knowledge; longing to return to the placement hospital to practice clinical skills; and acknowledgement of the importance of basic sciences in clinical work. Fluctuating trends (mainly showing a downward trend in academic year 2014/2015) were observed in statements related to prior clear plans to accomplish placement objectives; adequacy of variety and number of patients seen; adequacy of variety of clinical services at the placement hospital; supportive learning environment; utilization of planned time at the placement hospital; and being happy to have had a chance to utilize clinical skills. The one aspect, which showed a declining trend, was that related to hospital administration and staff being open and welcoming.

On attitudes towards peripheral hospital placement, analysis of individual constructs demonstrated an increasing trend only with regard to the construct that “Placing students in the peripheral hospital for clinical care is a good idea”. Insignificant fluctuating trends were observed with regard to liking the idea of peripheral hospital placement; having favourable attitudes towards peripheral hospital placement and likelihood to accept deployment to rural hospitals after completion of medical education.

Table 1 shows in-depth analysis according to constructs with respect to satisfaction with clinical practice competencies/skills acquired and satisfaction with preceptors/supervisors at the peripheral hospital during the three-year period. The findings revealed an increasing trend with regard to enjoyment in practicing and gaining new clinical clerkship skills at the peripheral hospital. Fluctuating trends were noted on issues regarding ease of practicing clinical skills and importance of teaching clinical skills at the peripheral hospital.

**Table 1:** Strength of consensus measure on aspects related to clinical practice competencies/skills acquired and satisfaction with preceptor/supervisor at the peripheral hospital.

Statement	2013/2014			2014/2015			2015/2016		

	n	Mean (SDev)	sCns (%)	n	Mean (SDev)	sCns (%)	n	Mean (SDev)	sCns (%)

**Clinical practice competencies/skills acquired at the peripheral hospital**									

I enjoyed practicing in the peripheral hospital	110	4.2 (0.8)	83.5	107	4.2 (0.7)	83.6	105	4.2 (0.7)	84.9
I found it easy to practice clinical skills in the peripheral hospitals	110	3.8 (0.9)	74.4	107	4.0 (0.8)	80.0	105	4.0 (0.9)	79.2
It was worthwhile teaching clinical practice in the peripheral hospital	110	4.2 (0.8)	83.6	107	4.1 (0.7)	82.8	105	4.3 (0.7)	85.4
I learnt a lot on clinical clerkship skills in the peripheral hospital	110	4.2 (0.8)	83.6	107	4.3 (0.6)	86.3	105	4.4 (0.6)	88.4
Mean sCns			81.3			83.2			84.5
**Satisfaction with preceptor/supervisor at the peripheral hospital**									

There was adequate interaction with preceptors/supervisors	109	4.1 (0.7)	80.9	107	4.2 (0.7)	84.1	104	4.0 (0.7)	80.5
The log books were reviewed regularly by our preceptors/supervisors	110	2.7 (1.2)	47.0	106	2.7 (1.1)	48.3	104	2.5 (1.2)	44.0
I received timely, constructive feedback on performance from my preceptors/supervisors	110	3.9 (0.9)	76.5	107	4.0 (0.8)	78.9	105	3.9 (0.8)	77.9
Mean sCns			68.1			70.4			67.5

Though agreement on adequacy of interaction with preceptors/supervisors showed a slight fluctuating trend during the three-year period, the strength of consensus measure was consistently above 80%, indicating strong agreement that the interaction with preceptors/supervisors was adequate. Very weak agreement was demonstrated on the aspect of regular review of logbooks by preceptors/supervisors (sCns < 50% for all three cohorts). Agreement on receiving timely and constructive feedback from preceptors/supervisors was also weak but sCns was above 76% for all three cohorts.

Comparison between students placed in rural and urban hospitals showed that attitude towards peripheral hospital placement was significantly positive for students placed in urban-located hospitals in all four constructs investigated, and in the adequacy of variety and number of clients seen (p < 0.05). However, students placed in rural-located hospitals were significantly satisfied with respect to administration and staff at the peripheral hospital being open and welcoming, and preceptors/supervisors regularly reviewing their logbooks (p < 0.05). Other constructs in the three domains did not differ significantly according to location of the peripheral hospital (p > 0.05).

**Figure 3:**
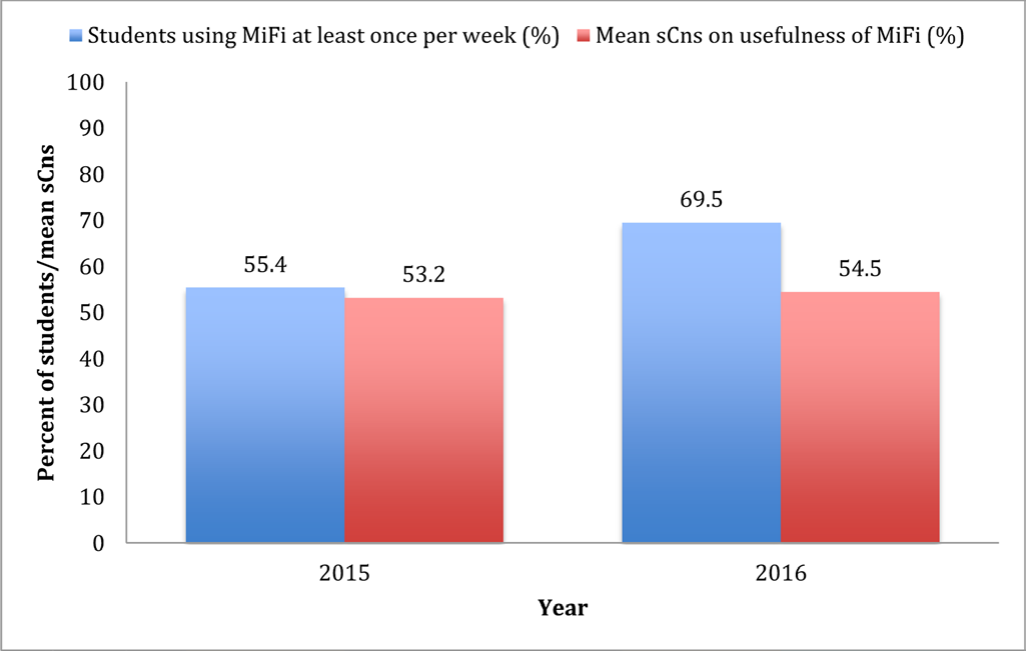
Frequency of use and mean consensus on usefulness of MiFi. sCns = Strength of consensus measure.

Table 2 shows the challenges/suggestion for improvement during the 2014 and 2015 rotations and actions taken to mitigate them. We analysed the effect of provision of Rapid Diagnostic Test (RDT) toolkit to the pilot hospitals on the satisfaction of students with and attitude towards peripheral hospital placement by comparing students who received the toolkits and those who did not. Significant differences (p < 0.05) in the satisfaction were observed in favour of students provided with toolkits, mainly in the general satisfaction with the rotation and ease of practicing clinical skills in the peripheral hospital. The proportion of students using MiFi at least once per week increased from 55.4% for the 2015 cohort to 69.5% for the 2016 cohort. On the other hand, the mean strength of consensus measure on the usefulness of MiFi almost remained stable between the two cohorts (sCns = 53.2% and 54.5% respectively) (Figure 3).

**Table 2:** Challenges/suggestion for improvement after peripheral hospital rotation.

Academic Year	Challenges/Suggestions	Action taken to resolve

2013/2014	1. Inability to apply knowledge gained during laboratory training and carry out point of care testing	Provision of RDT’s toolkit to one urban and one rural hospitals as pilot with wet laboratory personnel in each hospital for one week to assist the students during the laboratory sessions
	2. Availability of Internet services	Provision of MiFi to all hospitals
	3. Select hospitals with adequate number of clients	Difficult to implement due to fewer nearby hospitals that meet the selection criteria
	4. Initial planning and communication with peripheral hospitals	Initial arrangements on arrival of students for rotation were made by paying visits to respective hospitals

2014/2015	1. Provision of RDT’s toolkit to all hospitals	All teams in different hospitals were provided with RDT’s toolkit with wet laboratory personnel in each hospital for one week to assist the students during the laboratory sessions
	2. College to facilitate payment of internet bundle	MiFi were distributed to all teams with internet bundle
	3. Select hospitals with adequate number of clients	Difficult to implement due to fewer nearby hospitals that meet the selection criteria
	4. College supervisors should make regular assessment on the conduct of the hospital	Resident doctors from each of the major departments (surgery, paediatrics, internal medicine and obstetrics and, aecology) were rotating in each of the peripheral hospitals and for distant hospitals (Same, Gonja, Mt. Meru and St. Elizabeth), residing at the site.

## Discussion

Our study has shown that the majority of MD3 students were satisfied with the clinical rotations in peripheral hospitals, similar to findings reported in other studies [18,19]. Detailed analysis of trends of individual domains in our study showed that satisfaction with clinical practice competencies/skills acquired and attitudes toward peripheral hospital placement increased in consecutive cohorts (sCns > 80%), indicating strong agreement. Specifically, there was an increasing trend in the constructs of enjoyment of the placement, learning new clinical clerkship skills, and belief that it was a good idea to place students in peripheral hospitals.

Previous work has shown that peripheral hospital placement provides a chance for medical students to learn and practice clinical procedures by integrating basic sciences, clinical knowledge and skills into practice in the provision of patient care [20,19]. Our observations are also consistent with findings reported by Wilson et al. [21] where the majority of students reported enjoying their rural rotations, and that they acquired important procedural skills, and the rural experience enhanced their theoretical knowledge. Couper [22] and Mc Neill & Campbell [23] argued that successful rural placements offer greater opportunity to learn and practice procedures than at metropolitan teaching hospitals.

Regarding general satisfaction with the placements, increasing trends through the years were related to provision of clear initial orientation prior to placement; learning opportunities being consistent with learning goals; availability of opportunities to apply basic science knowledge; longing to return to the placement hospital to practice clinical skills; and acknowledgement of the importance of basic sciences in clinical work; most of them with strong agreement (sCns > 80%). It has been suggested that successful rural placements must give consideration to the interaction between learning needs, social needs and academic accountability [24] and also provide a supportive clinical environment [25]. Furthermore, Hampshire [26] attested that rural clinical placements link theory with clinical practice, thus offering experience in patient interviewing skills (communication skills).

Interactions of medical students during peripheral hospital placement with preceptors/supervisors were felt to be adequate with strong agreement (sCns > 80%). It has been argued that adequate supervision and feedback from supervisors/preceptors boosts the likelihood of the students toward peripheral rotations by assisting them to acquire the desired clinical competencies [27]. Andrews and Roberts [28] emphasize the importance of the relationships between staff and students. Hsue, Wilkson and Bills [29] point out that students learn and gain skills/competencies through interpersonal relationships between preceptors and students since preceptors are mentors and role models and also influence student career choices [29,30].

However, our study revealed a low strength of consensus measure with regard to timely and constructive feedback from supervisors/preceptors, and also regular review of logbooks. A study in Mwanza, Tanzania, it was found that only 75% of the students filled their logbooks during clerkship rotations, indicative of either lack of commitment on the part of students or inadequate supervision [31]. Causby [30] attests that positive feedback and advice from preceptors/supervisors’ influences student satisfaction with their clinical placements.

Attitudes towards peripheral hospital placements and adequacy of variety and number of patients seen were significantly positive for students placed in urban-located hospitals compared to rural-located hospitals. This could have been attributed to inadequacy of learning resources, including shortage of qualified preceptors, variety of patients, erratic availability of medical supplies and equipment, educational infrastructure such as teaching aid, libraries and access to information and communication technology in rurally located compared to urban-located peripheral hospitals. Other issues which impact student experiences include availability of comfortable accommodation and places for recreation in rural settings. Burch & Reid [32] argued that for rural clinical placements to be successful, some major challenges such as availability of teaching space in clinics to accommodate students, and heavy workloads for the few available healthcare workers must be addressed. Strasser & Neusy [33] also acknowledge the resource limitation challenge in rural hospitals, especially with regard to the shortage of teaching staff with the required clinical and teaching skills, poor quality of health services, and lack of good accommodation for medical students and educational resources. A conducive teaching and learning environment has been considered crucial during clinical clerkship rotations [34,8]. Availability of such conducive environments should result in positive attitudes toward peripheral hospital placement and facilitate student learning [35].

On the other hand, students in rural hospitals were significantly satisfied with respect to administration and staff at the peripheral hospital being open and welcoming, and preceptors/supervisors regularly reviewing their logbooks, even more frequently than their counterparts in urban hospitals. It has been shown that a student’s positive placement experience is influenced by relationships among supervisors/preceptors, hospital staff and other students during rotations [36]. However, rural hospitals are usually small in size with fewer health workers compared to urban hospitals, and hence health workers are more likely to have closer contact and relationships. Furthermore, students usually assist in service provision to ease the burden of fewer available health workers in rural hospitals [37] and also contribute to continuing education of the general practitioners at the host hospital, particularly rural hospitals [37].

Suggestions for improving clinical clerkship rotations mentioned by the students included increasing pocket money during the clinical rotation period, providing laboratory skills training at the clinical clerkship rotation hospitals, and provision of Internet services. Similar suggestions have also been reported in other studies [8,35]. These are viewed as important ingredients for creating conducive learning environment during peripheral hospital clinical clerkships.

In the context of LMICs, the clerkship rotations are positively viewed as a solution to reduce overcrowding at tertiary teaching hospitals, reducing the rural-urban imbalances of medical professionals by motivating medical students to choose rural practice and in some cases, reducing the burnout of medical personnel at the usually under-staffed peripheral hospitals. However, these rotations are not without drawbacks – such as costs that include accreditation of peripheral hospitals to provide quality learning environment, compensation to preceptors, providing medical supplies that may not be available at the practicing hospital to enable students to learn, transport costs for students and supervisors, accommodation costs and pocket money for upkeep of students. Also, due to varying contexts at different peripheral hospitals, including differences in equipment and medical supplies, availability of qualified staff to serve as preceptors and mentors, variations in the training and learning during rotations may vary among students of the same course-year. All these factors must be weighed before deciding to initiate clinical clerkships at peripheral hospitals.

### Limitations

- There was no control group of students who remained at KCMUCo for their junior clerkship practice to compare with those who practiced at the peripheral hospitals.
- A repeated cross-sectional study design (three different cohorts in different years) was used which only measures the aggregated change over time and thus is difficult to establish causality.
- The study depended on self-reports from students and were not objectively measured; hence the pertinent weakness of bias may be a factor. However, since we used strength of consensus measure to evaluate perceptions and attitudes towards peripheral hospital placement, we objectively assessed group agreement and hence reduced bias.

## Conclusion

Overall student experiences in peripheral clerkship rotations were positive with high level of student satisfaction. Opportunities exist for medical schools in sub-Saharan Africa to enhance medical training through the use of peripheral hospitals. From our experience careful implementation with elements of quality improvement and the inclusion of stakeholders is critical. Regular and timely feedback after each rotation serves as an important quality assessment tool for improvement of subsequent clinical clerkship rotations.

### Lessons learnt

- It is vital to listen to student voices and act upon their suggestions to improve their satisfaction with clinical rotations in peripheral hospitals by creating good learning environments.
- Proper on-site pre-assessment of host peripheral hospitals using a multi-disciplinary team and checklist designed based on the curriculum is important in order to objectively select hospitals that are capable of providing a good learning environment during clinical clerkships.
- Providing equipment and supplies that are not available/scarce at the placement hospital that required by the students for learning is important because firstly it enhances student learning, secondly to support the hospital in needed supplies/equipment and lastly, to enhance mutually beneficial relationships.
